# Finite element analysis of patient specific three-dimensional titanium plate versus conventional two miniplate fixation for mandibular angle fractures

**DOI:** 10.1186/s12903-026-09471-4

**Published:** 2026-08-08

**Authors:** Asmaa M. Abd Elfattah, Mostafa M. Eldibany, Ragab S. Hassan, Mona S. Oraby

**Affiliations:** Department of Oral and Maxillofacial Surgery, Faculty of Dentistry, Alexandria University, Alexandria, Egypt

**Keywords:** Mandibular angle fracture, Finite element analysis, Patient specific plate, Three-dimensional fixation, Titanium miniplate, Biomechanics

## Abstract

**Background:**

Mandibular angle fractures are among the most common maxillofacial fractures and represent a biomechanically challenging region because of complex functional loading. Conventional two miniplate fixation is widely used to provide fracture stability; however, stress concentration within the fixation hardware may affect its biomechanical performance. Recent advances in computer-aided design and manufacturing (CAD/CAM) have enabled the development of patient specific fixation techniques designed to improve implant adaptation and optimize stress distribution.

**Objective:**

This study aimed to compare the biomechanical behaviour of a patient specific three-dimensional (3D) titanium plate and conventional two miniplate fixation for mandibular angle fractures using finite element analysis (FEA).

**Materials and methods:**

A three-dimensional mandibular model was reconstructed from computed tomography data obtained from a 25-year-old fully dentate male patient. Cortical and cancellous bone structures were generated using Mimics and Geomagic Design X software. Two fixation models and fixation hardware were assembled using SolidWorks software: Model A consisted of conventional two miniplate fixation, while Model B consisted of a patient specific three- dimensional titanium plate contoured along the tension zone of the mandible. The models were imported into ANSYS software for finite element analysis under simulated unilateral clenching conditions. Von Mises stress, maximum principal stress, and total displacement were evaluated under identical loading and boundary conditions.

**Results:**

The patient specific 3D plate model demonstrated lower maximum von Mises stress within the fixation plate (326.19 MPa) compared with the conventional two miniplate system (417.48 MPa). Maximum principal stress in cortical bone was higher in the patient specific plate model (121.03 MPa) compared with the conventional fixation model (87.759 MPa). Total displacement was greater in the patient specific plate model (157.32 μm) than in the conventional system (131.47 μm). However, displacement values in both models remained within reported ranges considered acceptable for fracture healing.

**Conclusion:**

Patient specific and conventional fixation techniques exhibited distinct biomechanical characteristics under simulated loading conditions. Both implant design and fixation configuration influence the biomechanical behaviour of mandibular angle fracture fixation.

**Supplementary Information:**

The online version contains supplementary material available at 10.1186/s12903-026-09471-4.

## Introduction

Mandibular fractures are among the most common maxillofacial injuries, with fractures of the angle accounting for approximately 23–42% of all mandibular fractures. The high incidence of angle fractures is attributed to the unique anatomical and biomechanical characteristics of this region, including the transition between the mandibular body and ramus, reduced cross sectional bone mass, and the frequent presence of impacted third molars, all of which contribute to structural weakness and increased susceptibility to fracture [1].

Management of mandibular angle fractures remains challenging. Functional masticatory loading generates complex tensile, compressive, bending, and torsional stresses across the fracture site. In addition, the coordinated action of the masseter, temporalis, and medial pterygoid muscles contributes to fragment displacement and rotational instability. Therefore, the primary objective of treatment is to restore anatomical form and function while providing sufficient mechanical stability to permit functional loading and promote bone healing [1, 2].

Current concepts in mandibular fracture management encompass a variety of fixation techniques based on the principles of Champy osteosynthesis, including conventional miniplates, locking miniplates, three-dimensional plates, reconstruction plates, and patient specific fixation techniques [3]. According to Champy’s principles, single superior border miniplate fixation provides adequate stability for favourable mandibular angle fractures while minimizing surgical exposure and periosteal stripping [4]. Ellis similarly reported that a single miniplate placed along the external oblique ridge is sufficient for noncomminuted favourable angle fractures [5]. However, this technique may permit inferior border gapping, rotational instability, and reduced resistance to functional loading in displaced or unfavourable fracture patterns [6].

To improve biomechanical stability, several alternative fixation techniques have subsequently been developed. Conventional two miniplate fixation stabilizes both the superior tension zone and the inferior compression zone, thereby increasing fixation rigidity and reducing interfragmentary displacement under functional loading [6, 7]. Similarly, three-dimensional and matrix plate systems improve structural rigidity through interconnected plate designs that distribute functional loads more efficiently [8]. These fixation methods have shown favourable clinical and biomechanical outcomes in selected fracture patterns. However, they generally require greater surgical exposure and additional fixation hardware. Repeated plate bending may also alter the mechanical properties of titanium and increase operative time Consequently, no single fixation technique has demonstrated consistent superiority, and the choice of fixation method requires a balance between biomechanical stability, surgical invasiveness, implant adaptation, and operative complexity [9].

Recent advances in computer-aided design and computer-aided manufacturing (CAD/CAM) have facilitated the development of patient specific titanium fixation plates tailored to individual mandibular anatomy. These customized implants are designed directly from computed tomography data. They eliminate the need for intraoperative contouring while improving implant adaptation, optimizing screw trajectories, and avoiding critical anatomical structures such as the mandibular canal and dental roots [10, 11]. Liu et al. reported that topology-optimized patient specific titanium plates produced more favorable stress distribution and reduced stress shielding than conventional fixation techniques [12]. Whereas Saleh et al. demonstrated the clinical feasibility of CAD/CAM customized fixation, reporting improved implant adaptation and a more efficient surgical workflow [13]. These studies indicate that anatomical customization may improve implant adaptation and influence the biomechanical performance of mandibular fixation techniques.

Finite element analysis (FEA) has become an established computational method for investigating the biomechanical behaviour of biological structures [14]. By constructing three-dimensional models from medical imaging data and assigning appropriate material properties, FEA simulates physiological loading conditions to predict stress distribution, strain, deformation, and structural response. The technique has been widely applied in oral and maxillofacial surgery because it provides a non destructive, highly reproducible, and quantitative approach for evaluating fracture mechanics and the biomechanical performance of different fixation strategies [14, 15].

Previous finite element investigations have demonstrated that implant geometry and fixation configuration substantially influence stress distribution and fixation stability. Xu et al. experimentally validated a custom designed fixation plate and showed that implant design significantly influenced stress distribution and the mechanical behaviour of mandibular angle fracture fixation [15]. In addition, Subramanian et al. reported that different plate configurations generated distinct stress distribution patterns and displacement characteristics, highlighting the importance of implant design in achieving stable fracture fixation [16].

Despite these advances, evidence regarding the biomechanical performance of customized fixation techniques relative to established fixation strategies remains limited. In particular, whether a patient specific three-dimensional titanium plate based on the principles of superior border tension band fixation can achieve biomechanical performance comparable to that of the conventional two miniplate technique remains insufficiently investigated.

Therefore, the aim of this study was to compare the biomechanical performance of a patient specific three-dimensional titanium plate and conventional two miniplate fixation for mandibular angle fractures using finite element analysis.

## Materials and methods

### Study design

This comparative finite element analysis (FEA) study was conducted in accordance with the Declaration of Helsinki and approved by the Institutional Research Ethics Committee of the Faculty of Dentistry, Alexandria University (IRB No. 00010556102301/2025). Written informed consent was obtained from the participant before image acquisition.

A three-dimensional finite element model was reconstructed from computed tomography (CT) images of a 25-year-old male patient with a unilateral mandibular angle fracture and no craniofacial deformity or previous mandibular pathology. This patient was selected to minimise anatomical variables and represent normal adult mandibular biomechanics. CT images were acquired with a slice thickness of 1.0 mm and exported in Digital Imaging and Communications in Medicine (DICOM) format.

### Three-dimensional mandibular model reconstruction

**Fig. 1:**
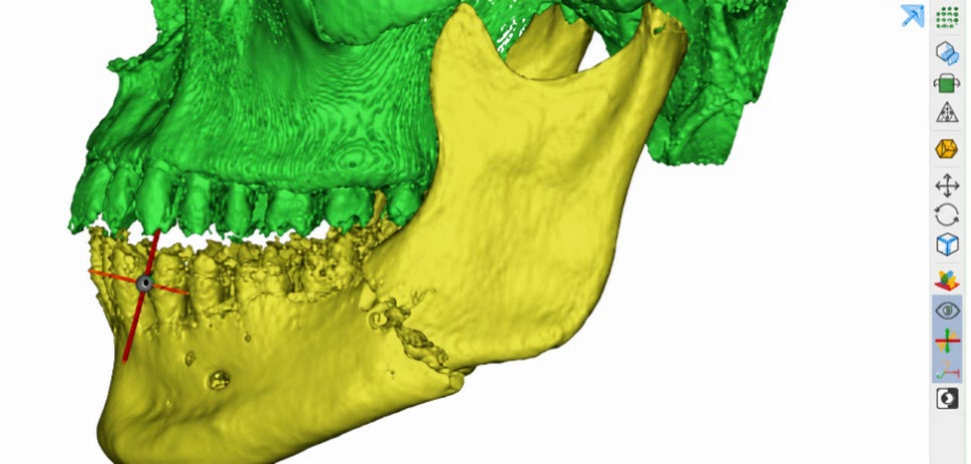
Three-dimensional mandibular reconstruction generated from computed tomography data following image segmentation

**Fig. 2:**
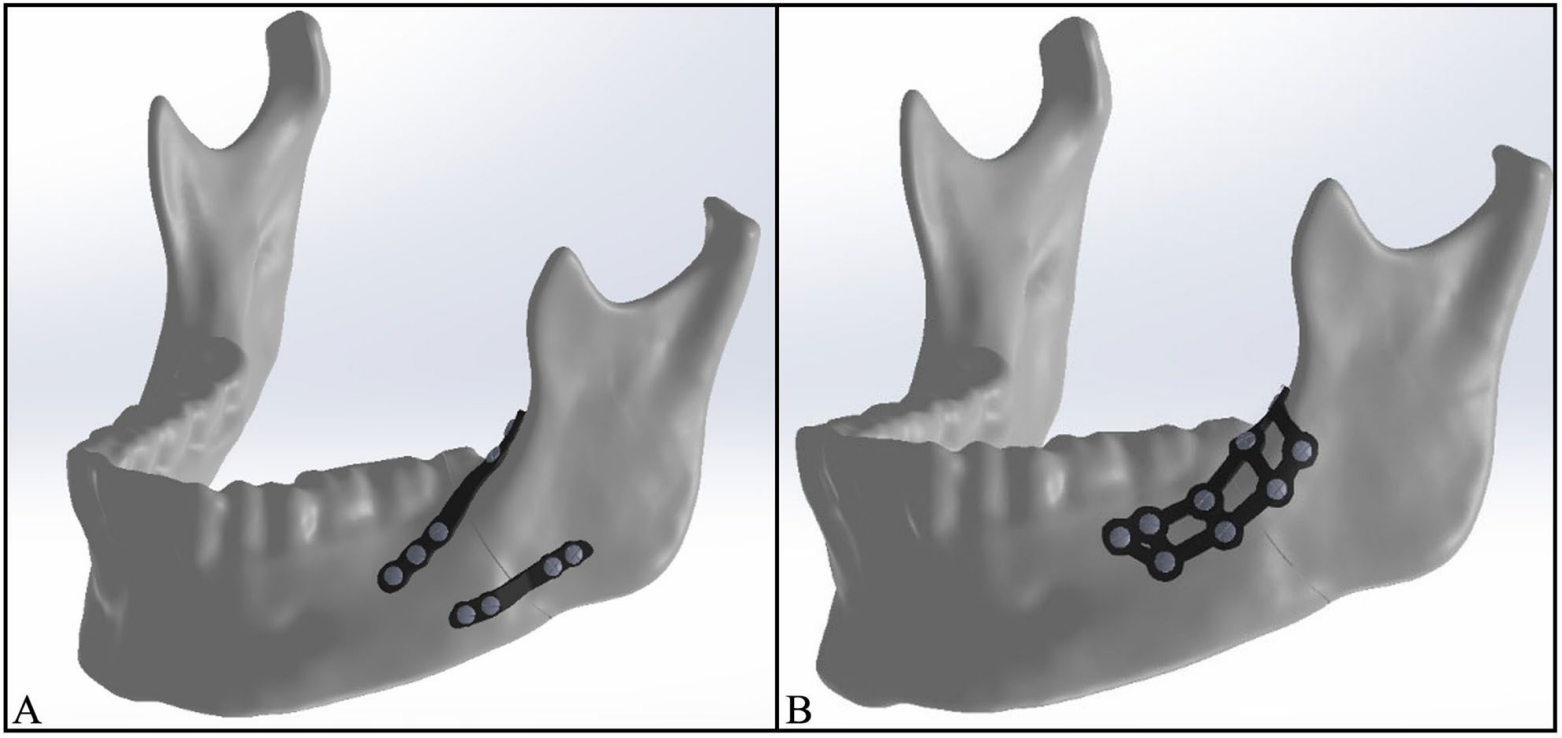
Three-dimensional mandibular fracture fixation models. **A** Conventional two miniplate fixation model with superior and inferior border plates. **B** Patient specific three-dimensional titanium plate fixation model positioned along the superior tension zone

The DICOM data were imported into Mimics Innovation Suite (Materialise NV, Leuven, Belgium) for image segmentation and three-dimensional reconstruction of the cortical and cancellous bone (Fig. 1). The reconstructed model was exported as a stereolithography (STL) file and imported into Geomagic Design X (3D Systems, Rock Hill, SC, USA) for surface smoothing, reverse engineering, and generation of a solid computer-aided design (CAD) model. Following virtual fracture reduction, the CAD model was subsequently imported into Solidworks (Dassault Systèmes, Waltham, MA, USA), where two fixation models were developed for biomechanical comparison (Fig. 2).

dfdf

#### Model A: Conventional two miniplate fixation

Model A comprised a conventional two miniplate fixation technique based on the Synthes Craniomaxillofacial (CMF) 2.0 system. One miniplate (5 screw holes) was positioned along the superior tension zone and the second miniplate (4 screw holes) along the inferior border of the mandible. Both miniplates were fixed using monocortical titanium screws (2.0 mm in diameter and 7 mm in length), modelled according to the manufacturer’s specifications.

#### Model B: Patient specific three-dimensional titanium plate fixation

Model B comprised a patient specific three-dimensional titanium plate fixation. The plate incorporated nine screw holes (five superior and four inferior) interconnected by transverse bars to form a single fixation unit. Screw positions and inter-hole spacing were individually adjusted to maximise the available bone stock while maintaining safe distances from the mandibular canal and adjacent dental roots. The plate had a uniform thickness of 1.0 mm and was fixed using the same monocortical titanium screws (2.0 mm in diameter and 7 mm in length) as those used in Model A.

### Finite element analysis

The completed fixation models were imported into ANSYS Workbench 2024 R2 (ANSYS Inc., Canonsburg, PA, USA) for finite element analysis. The cortical bone, cancellous bone, titanium plates, and screws were assumed to be homogeneous, isotropic, and linearly elastic. The material properties, including Young’s modulus and Poisson’s ratio, were obtained from previously published studies [12, 17, 18] and are summarised in Table 1.

**Table 1:** Material properties of each component of the model

	Modulus of elasticity (MPa)	Poisson’s ratio
Compact bone	13,700	0.3
Cancellous bone	142	0.3
Titanium for screw	114,000	0.33
Titanium for plate	116,000	0.34

**Fig. 3:**
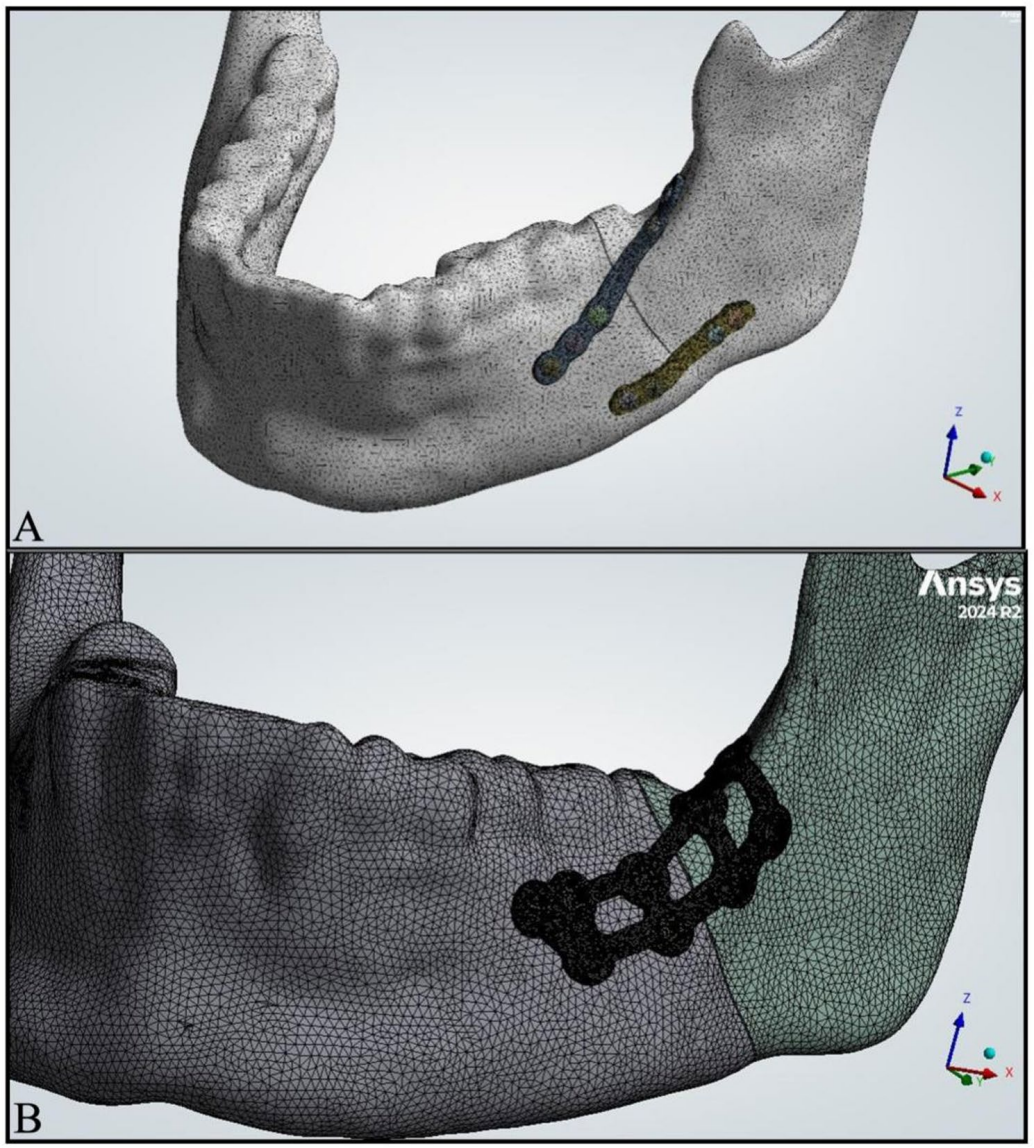
Finite element mesh of the mandibular fixation models showing discretisation into quadratic tetrahedral elements. **A** Conventional two miniplate fixation technique. **B** Patient specific three-dimensional titanium plate fixation technique

The finite element models were discretized using an unstructured mesh of quadratic tetrahedral elements (Fig. 3). The total numbers of nodes and elements for each model are presented in Table 2.

**Table 2:** The total number of elements and nodes for each model

	Nodes	Element
conventional Two miniplate	3,362,170	2,334,748
Patient specific plate	4,905,832	3,432,127

Physiological masticatory muscle forces were applied to the mandible to simulate unilateral clenching under static conditions. The applied forces were based on previously published biomechanical studies and were represented as concentrated vector loads acting at anatomically relevant muscle attachment site as follow [13]:

- Muscle forces of Temporalis (329.2 N).
- Masseter (272 N).
- Medial pterygoid (174.8 N).
- The forces applied in the anterior teeth region (62.8 N).
- The occlusal forces applied at the second molar teeth region (120 N).

Both mandibular condyles were constrained to simulate temporomandibular joint support during mandibular function. Fixed support was applied at the superior surfaces of the condylar heads, restricting translational movement in all directions while allowing rotational behavior. This boundary condition represents physiological joint stabilization during clenching and prevents rigid body motion of the mandible as shown in. (Fig. 3).

### Outcome measures

The biomechanical performance of both fixation models was evaluated by measuring von Mises stress, maximum principal stress, and total displacement. Stress and displacement distributions were displayed as color coded contour maps and quantified in megapascals (MPa) and micrometers (µm), respectively.

### Model validation

To validate the reliability of the developed finite element model, the total deformation values of the conventional two miniplate configuration were compared with published data from Subramanian et al. [16]. The predicted deformation in the present model (70.246 μm) closely approximated the reported value (71.92 μm), demonstrating acceptable agreement and confirming model validity for comparative biomechanical analysis.

## Results

Finite element analysis was performed to compare the biomechanical performance of Model A (conventional two miniplate fixation) and Model B (patient specific three-dimensional titanium plate) under identical loading and boundary conditions.

### Stresses distribution in fixation plates and screws

**Fig. 4:**
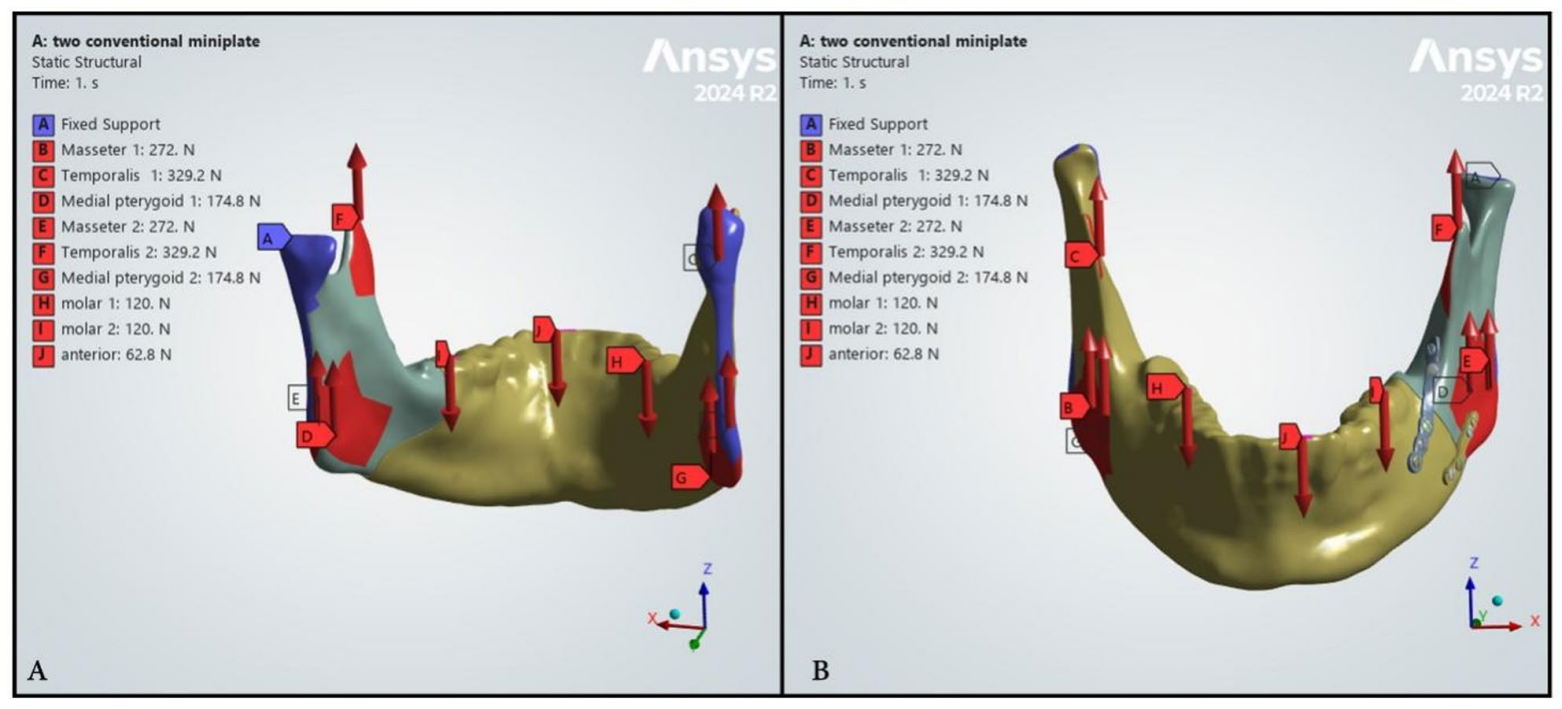
**A**, **B** Boundary conditions and loading application during finite element analysis demonstrating muscle force vectors and fixation of the mandibular condyles under simulated unilateral clenching

Model A exhibited a higher maximum von Mises stress within the fixation plates than Model B. The maximum plate stress was 417.48 MPa in Model A compared with 326.19 MPa in Model B (Fig. 4).

**Fig. 5:**
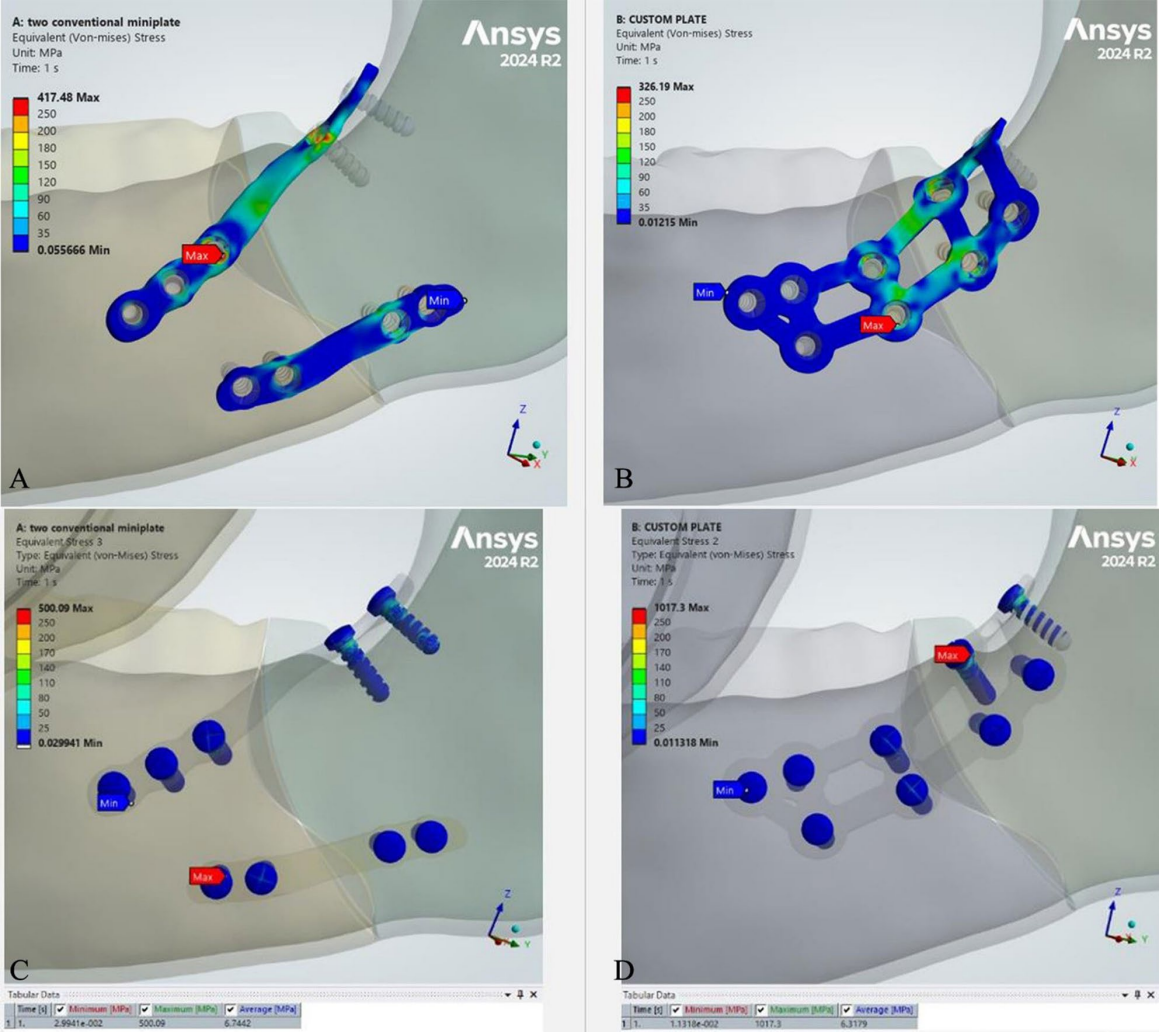
Von Mises stress distribution within fixation plates and screws. **A** Plate stresses in the conventional two miniplate system. **B** Plate stresses in the patient specific three-dimensional titanium plate system. **C** Screw stresses in the conventional two miniplate system. **D** Screw stresses in the patient specific three-dimensional titanium plate system

Similarly, the average von Mises stress in the fixation screws was slightly higher in Model A (6.7442 MPa) than in Model B (6.3179 MPa) (Fig. 5).

### Stress distribution in cortical bone

**Fig. 6:**
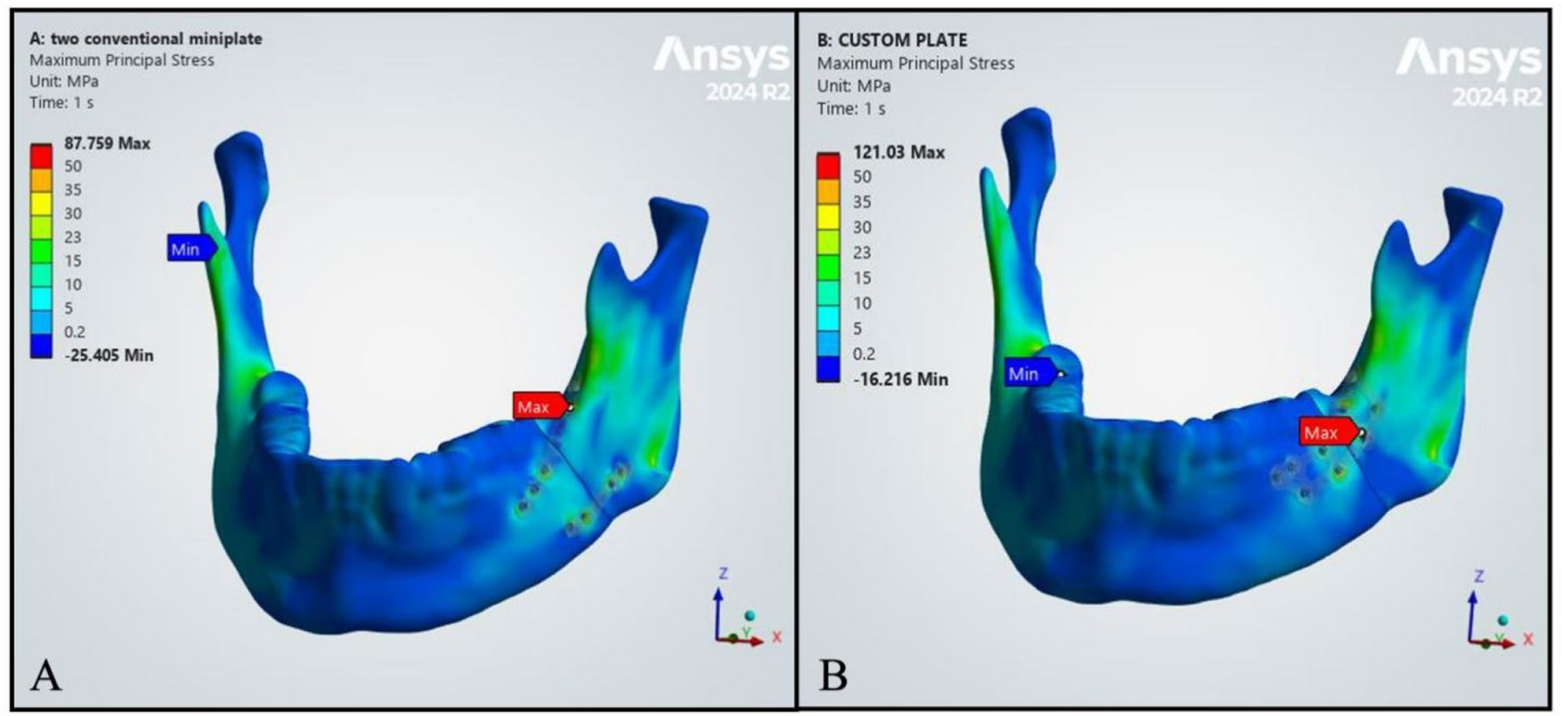
Maximum principal stress distribution in cortical bone surrounding the fracture site. **A** Conventional two miniplate fixation. **B** Patient specific three-dimensional titanium plate fixation

The bone is classified as a brittle material; hence the tensile & compressive stresses were applied. Maximum principal stress in the cortical bone was higher in Model B (121.03 MPa) than in Model A (87.759 MPa) (Fig. 6).

### Total displacement

**Fig. 7:**
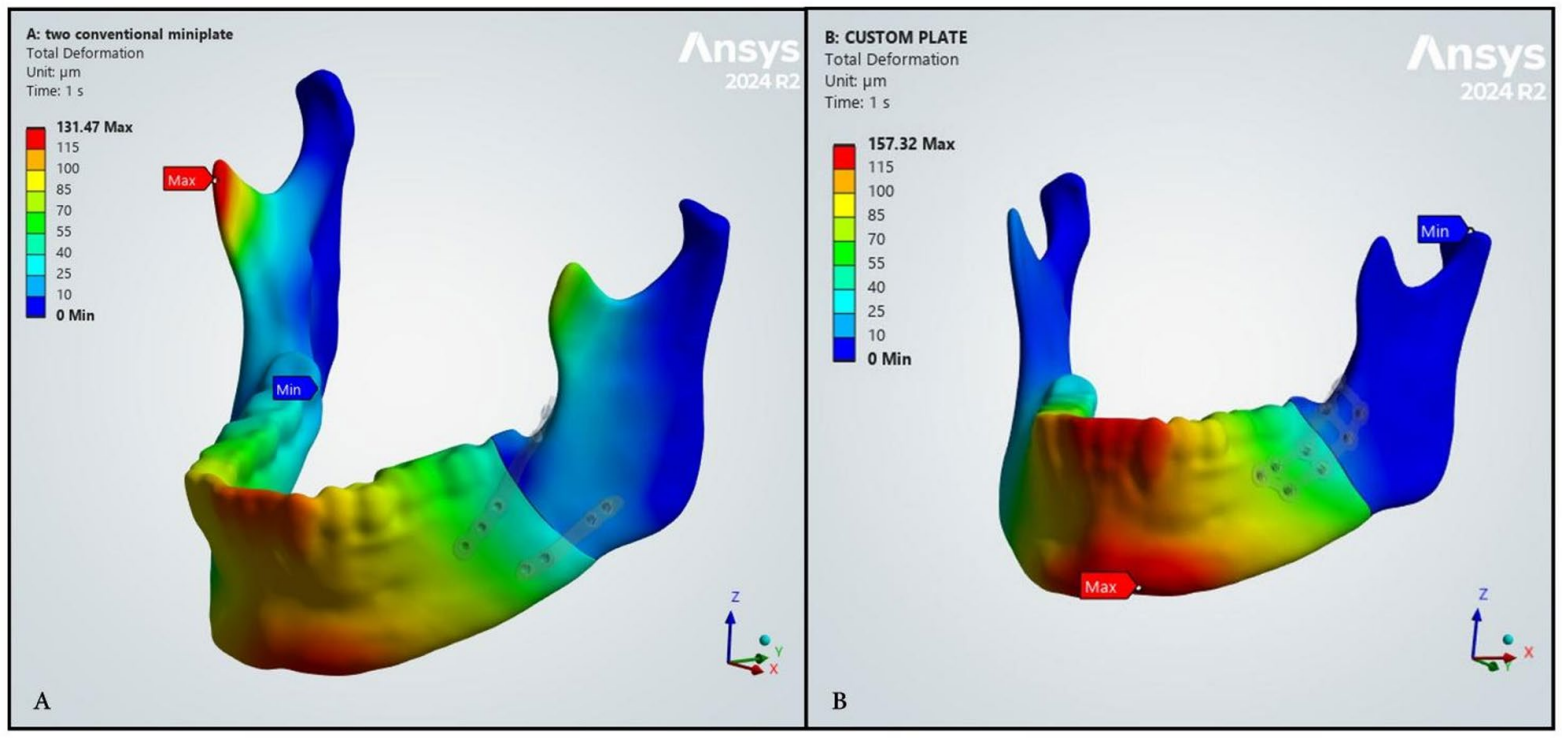
Total displacement distribution of the mandibular segments under functional loading. **A** Conventional two miniplate fixation technique. **B** Patient specific three-dimensional titanium plate fixation technique

Model B demonstrated greater total displacement than Model A. The maximum displacement was 157.32 μm in Model B and 131.47 μm in Model A (Fig. 7).

## Discussion

Stable fixation remains the principal objective in the management of mandibular angle fractures, as adequate mechanical stability is fundamental for fracture healing and early restoration of mandibular function. Finite element analysis has become an established tool for investigating the initial biomechanical behaviour of fixation strategies under standardized loading conditions and provides valuable insight into the mechanical performance of different fixation strategies before clinical application [14]. Therefore, the present study compared the biomechanical performance of a patient specific three-dimensional titanium plate and conventional two miniplate fixation for mandibular angle fractures.

According to the present study, the patient specific three-dimensional titanium plate exhibited lower von Mises stress than the conventional two miniplate fixation technique. This finding may be attributed to its customized geometry and precise anatomical adaptation, homogeneous load transfer and reduced stress concentration within the fixation device. Similar findings have been reported in previous biomechanical investigations. Lovald et al. reported that optimization of plate geometry significantly influences stress distribution and the mechanical behavior of mandibular fixation techniques [19]. Similarly, Pandya et al. reported more favorable stress distribution with three-dimensional strut plates and emphasized that plate geometry and screw distribution influence fixation biomechanics [20].

. The static yield limit for titanium is approximately 1000 MPa [21]. It is of significance that loads transmitted through plates during the healing phase should never exceed the limit of tensile strength of the material. Lower implant stress may contribute to reducing implant fatigue and would maintain sufficient structural stability during functional loading [22]. Furthermore, repeated bending during adaptation of conventional plates may induce excessive strain. This may create predetermined points of weakness that act as stress concentrators under physiological loading. Over time, this may increase the risk of stress fatigue and contribute to complications such as titanium plate fracture, corrosion, screw loosening, and bone resorption [20].

Higher maximum principal stress was observed in the surrounding cortical bone with the patient specific three-dimensional titanium plate than with the conventional two miniplate fixation technique. This finding suggests greater transfer of functional loads from the fixation device to the adjacent cortical bone, reflecting a more load sharing pattern of fixation. Li et al. reported that the geometry and thickness of patient specific titanium plates influence stress shielding around mandibular fractures [23], whereas Liu et al. showed that customised fixation technique modify stress transfer within the bone plate construct [12]. As the cortical bone stresses remained within the physiological range, the observed load sharing pattern may promote a physiological mechanical environment for bone remodelling during fracture healing [24].

The patient specific plate demonstrated greater interfragmentary displacement than the conventional two miniplate fixation technique (157.32 μm vs. 131.47 μm). This difference is more likely related fixation configuration than by customisation alone. The patient specific plate primarily stabilised the superior tension zone. In contrast, the conventional fixation technique stabilised both the superior tension and inferior compression zones, resulting in more rigid fixation. This interpretation is consistent with validated finite element and mechanical studies demonstrating that two-mini plate fixation provides greater fracture stability than superior border fixation alone under functional loading [25, 26]. Similarly, Naeini et al. reported that different fixation plate configurations generated distinct stress distribution and interfragmentary displacement patterns, emphasising the influence of fixation configuration on the biomechanical behaviour of mandibular angle fractures [27].

According to relevant studies, when the fracture gap was over 2 mm, it leaded to a poor healing [28]. The patient specific plate provided a less rigid fixation that permit a range of micromotion, but within the permissible limit for uncompromised bone healing [29]. The conventional two miniplate technique achieved greater fixation rigidity and decreased micromotion of fracture segments under the simulated loading conditions.

Overall, the present findings indicate that the investigated patient specific and conventional two miniplate fixation techniques exhibit distinct biomechanical characteristics, reflecting different approaches to fracture stabilisation. The patient specific fixation technique demonstrated a more load sharing pattern with lower implant stress, whereas the conventional two miniplate technique provided more rigid fixation with lower interfragmentary displacement. Neither biomechanical behaviour should be considered inherently preferable, as the optimal fixation strategy depends on fracture characteristics, treatment objectives, and patient specific factors. In addition to biomechanical performance, practical considerations such as anatomical adaptation, surgical accessibility, operative efficiency, implant availability, and surgeon experience may also influence the choice of fixation [10]. Accordingly, the choice of fixation should be guided by the intended biomechanical objective together with the clinical requirements of the individual patient.

Patient specific fixation may be considered when accurate anatomical adaptation without repeated plate bending and a functionally stable, load sharing fixation pattern are desired. Whereas conventional two miniplate fixation may be preferred when greater fixation rigidity is the primary objective. Nevertheless, these computational findings should be interpreted cautiously, as finite element analysis cannot fully replicate the complex biological processes involved in fracture healing.

The present study has several limitations. First, the finite element model was based on the mandibular anatomy of a single individual with one mandibular angle fracture pattern, which may not fully represent the anatomical variability, bone quality, and fracture characteristics encountered in clinical practice. This may limit the generalizability of the findings to all mandibular angle fractures. Second, the patient specific and conventional fixation techniques differed in both plate design and fixation configuration. Consequently, the observed biomechanical differences cannot be attributed solely to plate customization as differences in fixation configuration likely also contributed to the observed stress distribution and interfragmentary displacement. Third, the finite element analysis was performed under simplified modelling assumptions and static loading conditions, without incorporating cyclic fatigue, patient specific muscle activity, postoperative bone remodelling, or biological fracture healing. Therefore, although finite element analysis provides valuable biomechanical insight into the initial mechanical behaviour of fixation techniques, further experimental validation and prospective clinical studies are required before these findings can be translated into clinical practice. Future studies should also include additional fixation strategies, particularly single superior border miniplate fixation, to provide a more comprehensive biomechanical comparison of conventional and patient specific fixation techniques.

## Conclusion

Within the limitations of this study, finite element analysis showed that patient specific three-dimensional titanium plate fixation and conventional two miniplate fixation exhibit distinct biomechanical characteristics in the management of mandibular angle fractures. The patient specific plate reduced implant stress and exhibited a load sharing pattern of fixation, whereas the conventional two miniplate technique provided more rigid fixation and lower interfragmentary displacement. These findings suggest that the biomechanical behaviour of mandibular angle fracture fixation is influenced by both implant design and fixation configuration. However, further experimental validation and prospective clinical studies are required to determine the clinical significance of these biomechanical findings.

## Supplementary Information

Supplementary Material 1.

## Data Availability

The datasets used and/or analysed during the current study are available from the corresponding author upon reasonable request.

## Abbreviations

DICOM: Digital Imaging and Communications in Medicine
CAD/CAM: Computer aided design and Computer aided manufacturing
CT: Computed Tomography
FEA: Finite element analysis
3D: Three-dimensional
CMF: Craniomaxillofacial
VM: Von Mises
MPa: Megapascals
μm: Micrometers

## References

[1] Yadav S, Tyagi S, Puri N, Kumar P, Kumar P. Qualitative and quantitative assessment of relationship between mandibular third molar and angle fracture on North Indian population: Aclinicoradiographic study. Eur J Dent. 2013;7:2127. 10.4103/1305-7456.110188 PMC4023186 24883029

[2] Boffano P, Kommers SC, Karagozoglu KH, Gallesio C, Forouzanfar T. Mandibular trauma: a twocentre study. Int J Oral Maxillofac Surg. 2015;44:9981004. 10.1016/j.ijom.2015.02.022 25813086

[3] Champy M, lode JP, Schmitt R, Jaeger JH, Muster D. mandibular osteosynthesis by miniature screwed plates –Michelet technique II. J Oral Maxillofac Surg. 1978;6:1421. 10.1016/s0301-0503(78)80062-9 274501

[4] Singh R, Agrawal A, Pal U, Singh G. Single miniplate osteosynthesis in angle fracture. Natl J Maxillofac Surg. 2011;2:47. 22442609 10.4103/0975-5950.85853 PMC3304215

[5] Ellis E 3. Treatment methods for fractures of the mandibular angle. Int J Oral Maxillofac Surg. 1999;28:24352. 10416889

[6] Siddiqui A, Markose G, Moos KF, McMahon J, Ayoub AF. One miniplate versus two in the management of mandibularangle fractures: a prospective randomised study. Br J Oral Maxillofac Surg. 2007;45:2235. 10.1016/j.bjoms.2006.08.016 17110006

[7] Khemka G, Bhola N, Jadhav A, Borle R, Jain A, Dalmia S. What is a better modality of fixation in mandibular angle fractures, single miniplate or two miniplates? A singleblind comparative study. Natl J Maxillofac Surg. 2025;16:34753. 10.4103/njms.njms_10_24 PMC12469168 41019680

[8] Wusiman P, Yarbag A, Wurouzi G, Mijiti A, Moming A. Three-dimensional versus standard miniplate fixation in management of mandibular fractures: A systematic review and metaanalysis. J Craniomaxillofac Surg. 2016;44:164654. 10.1016/j.jcms.2016.07.027 27618717

[9] AlMoraissi EA, Ellis E 3rd. What method for management of unilateral mandibular angle fractures has the lowest rate of postoperative complications? A systematic review and metaanalysis. J Oral Maxillofac Surg. 2014;72:2197211. 10.1016/j.joms.2014.05.023 25236822

[10] Marschall J, Flint RL, Kushner GM, Alpert B, Azevedo B. In–house computer–aided design and manufacturing versus traditional open reduction and internal fixation for mandibular trauma. Oral Surg Oral Med Oral Pathol Oral Radiol. 2020;130:e23.

[11] Shehab MF, Taalab D, Abdel Rasoul MA. Evaluation of virtual surgical planning and CAD/CAM techniques in treatment of mandibular fractures via three-dimensional patient specific titanium plates: a case series. Egypt Dent J. 2021;67:277–84.

[12] Liu YF, Fan YY, Jiang XF, Baur DA. A customized fixation plate with novel structure designed by topological optimization for mandibular angle fracture based on finite element analysis. Biomed Eng Online. 2017;16:131. 29141673 10.1186/s12938-017-0422-z PMC5688740

[13] Saleh HO, Moussa BG, Salah Eddin KA, Noman SA, Salah AM. Assessment of CAD/CAM Customized V Pattern Plate Versus Standard Miniplates Fixation in Mandibular Angle Fracture (Randomized Clinical Trial). J Maxillofac Oral Surg. 2023;22:9951005. 10.1007/s12663-023-02027-x PMC10719228 38105847

[14] Sancar B, Çetiner Y, Dayı E. Evaluation of the pattern of fracture formation from trauma to the human mandible with finite element analysis. Part 2: the corpus and the angle regions. Dent Traumatol. 2023;39:437–47. 36942890 10.1111/edt.12841

[15] Xu X, Cheng KJ, Liu YF, Fan YY, Wang JH, Wang R, et al. Experimental validation of finite element simulation of a new customdesigned fixation plate to treat mandibular angle fracture. Biomed Eng Online. 2021;20:15. 33546713 10.1186/s12938-021-00851-1 PMC7866451

[16] Subramanian A, Rudagi BM, Londhe P, Palande C. A Finite Element Analysis of Single, Double & Matrix Miniplate in Fracture of the Mandibular ANGLE Region: An In Vitro Study. J Maxillofac Oral Surg. 2024;23:11421. 10.1007/s12663-022-01799-y PMC10830939 38312983

[17] Arantes VOR, Corrêa CB, Lunardi N, Boeck Neto RJ, SpinNeto R, Boeck EM. Insertion angle of orthodontic miniimplants and their biomechanical performance: finite element analysis. Rev Odontol UNESP. 2015;44:2739.

[18] Seo JH, Kim MS, Lee JH, EghanAcquah E, Jeong YH, Hong MH, et al. Biomechanical Efficacy and Effectiveness of Orthodontic Treatment with Transparent Aligners in Mild Crowding DentitionA Finite Element Analysis. Mater (Basel). 2022;15:3118. 10.3390/ma15093118 PMC9104142 35591454

[19] Lovald ST, Khraishi T, Wagner J, Baack B, Kelly J, Wood J. Comparison of platescrew systems used in mandibular fracture reduction: finite element analysis. J Biomech Eng. 2006;128:65462. 10.1115/1.2244575 16995751

[20] Pandya H, Patel H, Vithalani S, Bhavsar B, Shah U, Chunawala A. Finite Element Analysis of New Modified Three-dimensional Strut Miniplate versus Conventional Plating in Mandibular Symphysis and Angle Fractures An In vitro Study. Ann Maxillofac Surg. 2024;14:715. 10.4103/ams.ams_205_23 PMC11340833 39184404

[21] Niinomi M. Mechanical properties of biomedical titanium alloys. Mater Sci Eng A. 1998;243:2316.

[22] Gareb B, Roossien CC, van Bakelen NB, Verkerke GJ, Vissink A, Bos RR, et al. Comparison of the mechanical properties of biodegradable and titanium osteosynthesis systems used in oral and maxillofacial surgery. Sci Rep. 2020;10:18143. 33097757 10.1038/s41598-020-75299-9 PMC7584639

[23] Li Y, Li H, Lai Q, Xue R, Zhu K, Deng Y. Finite element analysis of 3Dprinted personalized titanium plates for mandibular angle fracture. Comput Methods Biomech Biomed Engin. 2023;26:7889. 10.1080/10255842.2022.2047952 35587215

[24] An YH, Draughn RA. Mechanical Testing of Bone and the Bone Implant Interface. 1st ed. London: CRC; 1999. p. 43.

[25] Daqiq O, Roossien CC, Wubs FW, van Minnen B. Biomechanical assessment of mandibular fracture fixation using finite element analysis validated by polymeric mandible mechanical testing. Sci Rep. 2024;14:11795. 38782942 10.1038/s41598-024-62011-4 PMC11116419

[26] Ayali A, Erkmen E. Biomechanical evaluation of different plating methods used in mandibular angle fractures with 3dimensional finite element analysis: favorable fractures. J Oral Maxillofac Surg. 2017;75:146474. 10.1016/j.joms.2017.02.028 28384464

[27] Naeini E, Sarkarat F, Jamilian A, Kalantar Motamedi MH. Comparative Evaluation of 7 Fixation Plates in Mandibular Angle Fractures Using Finite Element Analysis. Trauma Mon. 2020;25:14552.

[28] Claes L, Augat P, Suger G, Wilke HJ. Influence of size and stability of the osteotomy gap on the success of fracture healing. J Orthop Res. 1997;15(4):577–84. 10.1002/jor.1100150414. 9379268 10.1002/jor.1100150414

[29] Wang R, Liu Y, Wang JH, Baur DA. Effect of interfragmentary gap on the mechanical behavior of mandibular angle fracture with three fixation designs: a finite element analysis. J Plast Reconstr Aesthet Surg. 2017;70:3609. 10.1016/j.bjps.2016.10.026 27939907

